# Impact of Different CKD Definitions on Long-Term Renal Function and Mortality in a Population-Based Cohort Study

**DOI:** 10.1016/j.ekir.2024.11.027

**Published:** 2024-11-28

**Authors:** Delal Dalga, Aurélie Huber, Anne Dufey, Peter Vollenweider, Pedro Marques-Vidal, Sophie de Seigneux, Belen Ponte, Lena Berchtold

**Affiliations:** 1 Department of Medicine, Laboratory of Nephrology, University Hospitals of Geneva, Geneva, Switzerland; 2 Department of Cell Physiology, Faculty of Medicine, University of Geneva, Geneva, Switzerland; 3 Department of Medicine, Division of Nephrology and Hypertension, University Hospitals of Geneva, Geneva, Switzerland; 4 Department of Medicine, Internal Medicine, Lausanne University Hospital and University of Lausanne, Lausanne, Switzerland

**Keywords:** age, body surface area, chronic kidney disease, glomerular filtration rate, mortality prognosis

## Abstract

**Introduction:**

The adoption of age or individualized body surface area (i-BSA) estimated glomerular filtration rate (eGFR) thresholds could influence the prevalence and prognosis of chronic kidney disease (CKD). This longitudinal study with up to 15 years of follow-up in the general population, compares different eGFR thresholds for CKD definition: standard, corrected to i-BSA, and age-stratified. For each, we assessed the prevalence of CKD and the combined impact on rapid renal function decline (RRFD) and mortality.

**Methods:**

Patients were classified as CKD according to the presence of significant albuminuria and/or different eGFR thresholds as follows: (i) < 60ml/min per 1.73 m^2^; (ii) < 60ml/min corrected to i-BSA; (iii) stratified by age, that is, < 75, < 60 and < 45 ml/min per 1.73 m^2^ if aged < 40 years, 40 to 65 years, and > 65 years, respectively. We performed adjusted Cox regression analyses to predict RRFD and global mortality.

**Results:**

We analyzed 4952 participants (54% women; mean age: 52 years). Age-stratified definition resulted in 24 of 677 participants aged < 40 years reclassified as CKD, with no adverse outcomes; whereas 55 of 713 participants aged > 65 years were reclassified as non-CKD, with 12 deaths and 1 RRFD. After multivariate adjustment, the CKD group had a poorer prognosis compared with the non-CKD group independently of the definition used; hazard ratio (HR) and 95% confidence interval (CI) were 2.23 (1.59–3.12), 2.06 (1.46–2.90), and 1.64 (1.13–2.38) for the standard, corrected to i-BSA, and age-stratified definitions, respectively.

**Conclusion:**

In our study, classification of CKD by age or i-BSA does not appear to improve prediction of RRFD and mortality.

## Graphical abstract

**Figure:**
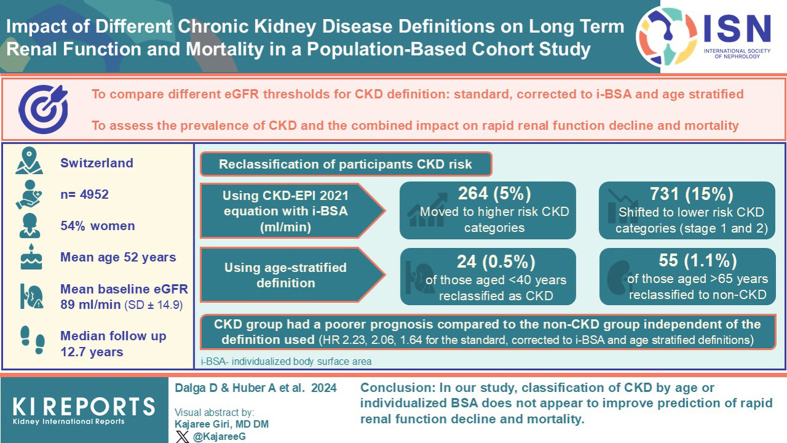

CKD affects millions of people worldwide, ranking as the 12th leading cause of mortality.^1^ CKD is defined as abnormalities of kidney structure or function, present for more than 3 months. Classification of CKD is based on the 2013 Kidney Disease Improving Global Outcomes guidelines,^2^ which incorporate glomerular filtration rate (GFR) categories and albuminuria levels to categorize CKD into 5 stages. However, these severity stages, as well as the 60 ml/min per 1.73 m^2^ cutoff have not been calibrated to age, even though kidneys change structurally and physiologically through the process of normal senescence, with a natural aging eGFR decline after 40 years.^3^

Based on the physiological aging process, one can postulate that using the same CKD-GFR threshold for everyone might not be relevant in predicting mortality across all ages categories. Delanaye *et al.* ^4^ conducted a secondary analysis of data from the CKD Prognosis Consortium to further explore the association between GFR and mortality across different age groups. The results indicated that in the 18 to 54 years age category, the mortality risk increases with an eGFR < 75 ml/min per 1.73 m^2^. In the 55 to 64 years age category, mortality risk becomes significant with an eGFR < 60 ml/min per 1.73 m^2^. Finally, for the group aged > 65 years, the mortality risk increases significantly with an eGFR < 45 ml/min per 1.73 m^2^. This study demonstrates how mortality risk increases at different eGFR ranges across various age categories. Therefore, the authors suggested redefining CKD by using age-adapted definitions based on percentiles of GFR from healthy populations. In a population-based cohort from Alberta, Canada, researchers showed that employing a fixed eGFR threshold led to a 60% increase in CKD prevalence, mainly among participants aged > 65 years. Interestingly, these individuals showed lower risks of kidney failure and death, comparable with rates observed in non-CKD cohorts.^5^ Thus, modifying the CKD definition to include age-specific eGFR thresholds instead of a fixed one could influence CKD prevalence and reduce both nonessential care and costs in an elderly population. Despite these studies, Kidney Disease Improving Global Outcomes 2024 guidelines have changed neither the CKD-GFR threshold definition nor the classification.

The use of individualized instead of standardized 1.73 m^2^ BSA for estimating renal function remains a subject of debate because it may better represent renal function for individuals with extreme weights. Delanaye *et al.*^6^ also explored this issue, applying i-BSA to eGFR thresholds, uncovering specific limitations of this approach in obese and anorexic individuals.

In the present study, we aimed to evaluate CKD reclassification within a Swiss general population cohort by applying the following 3 different equations: (i) the conventional fixed thresholds used in CKD-Epidemiology Collaboration (CKD-EPI) 2021 equation, (ii) i-BSA eGFR, and (iii) age-specific eGFR thresholds. We then studied their effects on the prediction of RRFD and mortality. We hypothesized that the use of age-specific or i-BSA criteria will result in a decreased CKD prevalence and a reduced mortality and RRFD, over and up to 15 years follow-up period.

## Methods

### Participants

The data set for this investigation was derived from the CoLaus/PsyCoLaus study, a general population-based cohort study in Lausanne, Switzerland, with follow-up assessments conducted at 5, 10, and 15 years.^7^

The baseline survey took part between 2003 and 2006, encompassing a cohort of 6733 individuals aged 35 to 75 years, of whom 6184 were Caucasian. Detailed methodologies concerning participant selection, evaluation protocols, and clinical data acquisition have been documented in a previous study.^7^ Briefly, recruitment occurred within the city of Lausanne, which had a population of 117,161 residents at that time. The municipal registry provided a complete list of 56,694 residents within the age range (35–75 years), from which a simple, nonstratified random sampling method was used to contact 35% of this population through mailed correspondence. The inclusion criteria stipulated Caucasian ethnicity and the ability to provide written informed consent. The response rate for this randomly selected sample was 41%. Patients with missing data on albuminuria and normal eGFR according to 1 of the 3 CKD definitions, or patients with no follow-up either at 5, 10, or 15 years were excluded. This study, involving human participants was reviewed and approved by the local ethics committee “Commission cantonale d’éthique de la recherche sur l’être humain” (CER-VD: VD-16/03; VD-33/09, and VD-26/14) and was conducted in accordance with the Declaration of Helsinki. The patients/participants provided their written informed consent to participate in this study.

### Survey and Clinical Data Acquisition

Trained health care professionals administered standardized questionnaires to collect data on socio-demographic and lifestyle factors. These surveys included items on tobacco and alcohol use, as well as treatment history. For this analysis, “smokers” refers to current smokers, and “nonsmokers” to those who have never smoked or are former smokers.

Body mass index (BMI) was determined using the formula: weight (in kg) divided by height squared (in m^2^). Subsequently, BMI was categorized as “normal” for BMI < 25 kg/m^2^, “overweight” for 25 ≤ BMI < 30 kg/m^2^, and “obese” for BMI ≥ 30 kg/m^2^.

Blood pressure measurements were taken 3 times on the left arm of seated participants using a clinically validated automatic oscillometric device.^8^ The mean of the final 2 readings were used to evaluate hypertension, which was defined as mean systolic blood pressure ≥ 140 mm Hg and/or a diastolic blood pressure ≥ 90 mm Hg, or the current use of antihypertensive medication.

Diabetes was identified as fasting blood glucose levels ≥ 7 mmol/l^9^ or the use of antidiabetic medication.

### Laboratory Data

Blood samples were obtained from participants after overnight fasting. The CHUV Clinical Laboratory conducted clinical chemistry assays on these samples. Serum and urine creatinine levels were measured using the IDMS-traceable Jaffe kinetic compensated method by Roche Diagnostics, Switzerland, which guarantees accuracy and reliability, with maximum intra- and inter-batch coefficients of variation ranging from 2.9% to 0.7%.

For albuminuria assessment, a random single morning spot urine specimen was collected. Immuno-nephelometry quantified the albumin levels. Albumin-to-creatinine ratio was calculated from the creatinine and albumin values.

### CKD Definition

GFR estimation was conducted using the CKD-EPI 2021 equations.^10^ Albumin-to-creatinine ratio values > 30 mg/g signified albuminuria, including both A2 (albumin-to-creatinine ratio: 30–299 mg/g) and A3 (albumin-to-creatinine ratio: ≥ 300 mg/g) albuminuria classifications.^11^, ^12^, ^13^ CKD was diagnosed based on either the eGFR thresholds defined below, or significant albuminuria.

We categorized patients by sequentially using 3 CKD-definitions, each including albuminuria yet distinguished by varying eGFR thresholds as follows: (i) eGFR < 60 ml/min per 1.73 m^2^; (ii) eGFR < 60 ml/min corrected to i-BSA; (iii) age-stratified eGFR with CKD defined as eGFR < 75 ml/min per 1.73 m^2^ for individuals aged < 40 years, < 60 ml/min per 1.73 m^2^ for those aged 40 to 65 years, and < 45 ml/min per 1.73 m^2^ for those aged > 65 years, according to the previous studies definitions.^14^

### Outcome Definition

The primary outcome was a combined incidence of RRFD and global mortality, defined as all-cause mortality, over the follow-up duration. Following the Kidney Disease Improving Global Outcomes guidelines, which defined RRFD as a sustained eGFR reduction of > 5 ml/min per 1.73 m^2^ per year,^15^ we defined RRFD as a decrease of > 25 ml/min per 1.73 m^2^ at 5 years, > 50 ml/min per 1.73 m^2^ at 10 years, and > 75 ml/min per 1.73 m^2^ at 15 years, comparing baseline value with follow-up measurement. These parameters were aggregated into a composite outcome to both represent the adverse progression that effective nephroprotection could potentially prevent and to increase the total number of events observed. The secondary outcomes were RRFD and mortality, analyzed separately.

### Statistical Analysis

Descriptive analysis presented continuous variables as mean ± SD or median with interquartile range, according to their distribution. Categorical variables were expressed as counts and percentages.

Separate models using the 3 CKD definitions, namely the classical CKD-EPI 2021, i-BSA CKD, and age-stratified CKD definition, were deployed to examine their influence on the primary composite outcome, which includes mortality and RRFD. Kaplan-Meier estimates were used for assessing survival probabilities, whereas Cox proportional hazards models analyzed time-to-event data. Survival curves were compared using the log-rank test. Cox models provided HRs, 95% CIs, and associated *P* values. The assumption of hazard proportionality was visually verified using Schoenfeld residuals (log-minus-log survival plots against time).

Selection of covariates in multivariable Cox analyses was based on the existing scientific literature to identify potential confounders. The Cox proportional hazards model included variables such as diabetes, sex (male or female), BMI, arterial hypertension, treated dyslipidemia, smoking, and previous cardiovascular events.

In secondary analyses, RRFD and global mortality were examined separately.

In addition, Kaplan-Meier survival analysis was used to evaluate the prognostic implication of different eGFR CKD thresholds in 2 age-defined patient subgroups. For individuals younger than 40 years, eGFR-strata were ≥ 75, 60 to 74, and < 60 ml/min per 1.73 m^2^; for those aged ≥65 years, eGFR-strata were ≥ 60, 45 to 60, and < 45 ml/min per 1.73 m^2^, allowing for a comparative assessment of survival outcomes across these eGFR thresholds.

Missing data were assumed to occur at random, and a variable was excluded if missing data exceeded 5%. Statistical analyses were performed using STATA 18 (StataCorp) and R (R Foundation for Statistical Computing). Statistical significance was set at *P* < 0.05, applying 2-tailed tests.

## Results

### Participant Characteristics

**Figure 1:**
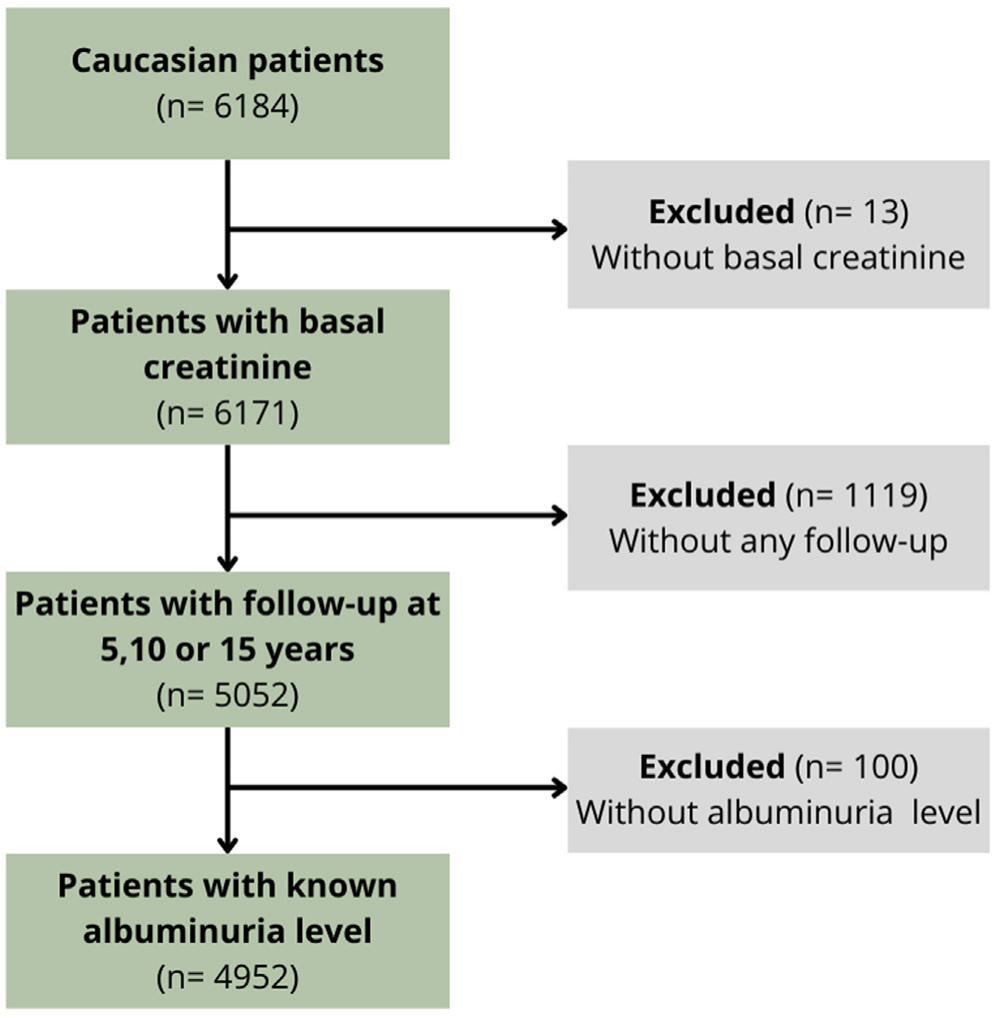
Flow chart presenting the studied group.

Among the 6184 Caucasian individuals of the CoLaus study, 4952 (80.1%) were included in our analyses. In Figure 1, we illustrate the study’s flowchart, and in Table 1, we provide the baseline characteristics of excluded and included participants. The exclusion criterion impacting the study the most was the absence of follow-up. When comparing excluded and included populations, mean eGFR, number of patients with a BMI between 25 and 30 kg/m^2^, and treated dyslipidemia were similar with no significant difference. Notably, the age distribution in the included population was narrower, with fewer very old and very young participants.

**Table 1:** Demographic and clinical characteristics differences between included and excluded patients

Characteristics	Included *n* (%)	Excluded *n* (%)	Statistical test (*P* value)^d^
N total (6184)	4952 (80.1)	1232 (19.9)	
Demographic characteristics			
Women	2670 (53.9)	581 (47.2)	<0.001
Age, median (IQR), yrs	51.8 (43.7–60.8)	55.25 (45.18–64.13)	<0.001
< 40 yrs	662 (13.4)	134 (10.9)	0.02
40–65 years	3598 (72.7)	822 (66.7)	<0.001
> 65 yrs	692 (14)	276 (22.4)	<0.001
BMI categories, kg/m^2^			
< 25	2442 (49.3)	527 (42.3)	<0.001
> 25–30	1805 (36.4)	447 (36.3)	0.92
> 30	705 (14.2)	256 (19.3)	<0.001
BSA, mean (SD), m^2^	1.87 (0.2)	1.85 (0.22)	
Laboratory findings			
eGFR^a^, mean (SD), ml/min per 1.73m^2^	88.9 (14.9)	88.8 (16.5)	0.98
Albuminuria^b^, median (IQR), mg/g	4.9 (3.3–8.7)	0.006 (0.003–0.01)	<0.001
Cardiovascular risk factors			
Diabetic^c^	288 (5.8)	119 (0.7)	<0.001
Hypertensive^c^	1673 (33.8)	26 (2.1)	<0.001
Treated dyslipidemia	527 (10.6)	186 (15.1)	0.48
Smoking	1289 (26)	384 (30.1)	<0.001
Prior cardiovascular events			
Myocardial infarction	182 (3.7)	25 (2.02)	<0.001
Angina pectoris	172 (3.5)	30 (2.4)	0.08
Heart failure	109 (2.2)	12 (0.9)	<0.001
Stroke	223 (4.6)	27 (2.2)	<0.001

BMI, body mass index; BSA, body surface area; CKD-EPI, chronic kidney disease-epidemiology collaboration; eGFR, estimated glomerular filtration rate; IQR, interquartile range.

a eGFR is calculated with CKD-EPI 2021 equation.

b Albuminuria was defined as albumin-to-creatinine ratio in mg/l.

c Baseline glycemia > 7 mmol/l or antidiabetic treatment. Hypertensive at baseline (> 140/90 mm Hg) or with hypertensive treatment.

d Statistical differences between the outcome groups were assessed using Pearson chi-square test for categorial variables, *t* test for continuous variable, with assessment of *P*-values.

The median follow-up duration was 12.7 years (SD: ± 2.9; range: 4.9–17.3). Within the cohort, 72 participants (1.4%) experienced RRFD and 170 (3.4%) died, of whom 11 (0.2%) experienced both outcomes. In Table 2, we present the details of the characteristics of participants with and without RRFD and mortality. The entire cohort was predominantly female (54%), mean age was 52 (interquartile range: 43.7–60.8) years, and mean baseline eGFR was 89 ml/min per 1.73 m^2^ (SD: ± 14.9). The combined outcome group consisted mainly of males (57%) who were older, had higher BMI, greater BSA, lower eGFR based on CKD-EPI 2021 criteria, and a higher incidence of albuminuria. In addition, this subgroup had a higher presence of cardiovascular risk factors, including hypertension, diabetes, smoking, and dyslipidemia. Higher occurrences of cardiovascular events, such as myocardial infarction, stroke, angina pectoris and heart failure, were observed among in the composite renal outcome group.

**Table 2:** Baseline demographic and clinical characteristics of study participants, categorized by the presence or absence of the combined outcome (RRFD and mortality)

Characteristics	All participants *n* (%)	With combined outcome *n* (%)	Without combined outcome *n* (%)	*P* value^d^
*N* total	4952	229 (4.6)	4723 (95.4)	
Demographic characteristics				
Female	2670 (53.9)	98 (42.8)	2572 (54.5)	<0.001
Age, median (IQR), yrs	51.8 (43.7-60.8)	62.0 (55.8-70.1)	52.1 (43.4-60.3)	<0.001
< 40 yrs	662 (13.4)	7 (3.1)	655 (13.9)	<0.001
40–65 yrs	3598 (72.7)	122 (53.3)	3476 (73.6)	<0.001
> 65 yrs	692 (14.0)	100 (43.7)	592 (12.5)	<0.001
BMI categories, kg/m^2^				
< 25	2442 (49.3)	68 (29.7)	2374 (50.3)	<0.001
> 25–30	1805 (36.4)	100 (43.7)	1705 (36.1)	<0.001
> 30	705 (14.2)	61 (26.6)	644 (13.6)	<0.001
BSA, mean (SD), m^2^	1.8 (0.2)	1.9 (0.2)	1.8 (0.2)	<0.001
Laboratory findings				
eGFR^a^, mean (SD), ml/min per 1.73 m^2^	88.9 (14.9)	84.6 (18.1)	89.1 (14.7)	<0.001
KDIGO stage G1	2475 (49.0)	2271 (49.4)	149 (41.9)	
KDIGO stage G2	2433 (48.2)	2220 (48.3)	171 (48.3)	
KDIGO stage G3	139 (2.8)	102 (2.2)	34 (9.6)	
KDIGO stage G4	4 (0.1)	2 (0.04)	2 (0.6)	
KDIGO stage G5	1 (0.02)	1 (0.02)	0	
Albuminuria^b^, median (IQR), mg/g	4.9 (3.3–8.7)	42.8 (4.3–16.9)	11.6 (3.3–8.4)	<0.001
KDIGO stage A1	4673 (92.5)	4373 (93.6)	300 (6.4)	
KDIGO stage A2	257 (5.1)	207 (80.5)	50 (19.5)	
KDIGO stage A3	122 (2.4)	16 (72.7)	6 (27.3)	
Cardiovascular risk factors				
Diabetic^c^	288 (5.8)	40 (17.5)	248 (5.3)	<0.001
Hypertensive^c^	1673 (33.8)	143 (62.4)	1530 (32.4)	<0.001
Treated dyslipidemia	527 (10.6)	41 (17.9)	486 (10.3)	<0.001
Smoking	1289 (26.0)	73 (31.9)	1216 (25.7)	0.039
Prior cardiovascular events				
Myocardial infarction	182 (3.7)	24 (10.5)	158 (3.3)	<0.001
Angina pectoris	172 (3.5)	18 (7.9)	154 (3.3)	<0.001
Heart failure	109 (2.2)	15 (6.6)	94 (2.0)	<0.001
Stroke	223 (4.6)	35 (15.3)	188 (4.0)	<0.001

BMI, body mass index; CKD-EPI, chronic kidney disease-epidemiology collaboration; eGFR, estimated glomerular filtration rate; IQR, interquartile range; KDIGO, Kidney Disease Improving Global Outcomes; RRDF, rapid renal function decline.

a eGFR is calculated with CKD-EPI 2021 equation. KDIGO stage G1 (≥ 90 ml/min per 1.73 m^2^), G2 (60–90 ml/min per 1.73 m^2^), G3 (30–59 ml/min per 1.73 m^2^), G4 (15–29 ml/min per 1.73 m^2^), G5 (<15 ml/min per 1.73 m^2^).

b Albuminuria was defined as albumin-to-creatinine ratio in mg/g. KDIGO stage A1 (< 30 mg/g), A2 (30–300 mg/g), and A3 (> 300 mg/g).

c Baseline glycemia > 7 mmol/l or antidiabetic treatment. Hypertensive at baseline (> 140/90 mm Hg) or with hypertensive treatment.

d Statistical differences between the outcome groups were assessed using Pearson chi-square test for categorial variables and *t* test for continuous variable, with assessment of *P*-value.

### Revised CKD Classification Using Different CKD Definitions

Our analysis incorporated 3 CKD definition groups: the first using the standard CKD-EPI 2021 equation, the second using the CKD-EPI 2021 equation corrected to i-BSA, and the third applying age stratification to the CKD-EPI 2021 equation.

In Table 3, we present the details of the reclassification of participants’ CKD risk according to the CKD-EPI 2021 equation with i-BSA (ml/min). A total of 264 individuals (5% of the cohort) were moved to higher-risk CKD categories, whereas 731 participants (15%) were shifted to lower-risk categories (stage 1 and 2). Using age-stratified definition, 24 patients (0.5%) aged < 40 years were reclassified as having CKD and 55 patients (1.1%) of those aged > 65 years were reclassified from CKD to non-CKD.

**Table 3:** Reclassification of participants CKD risk according to the CKD-EPI 2021 equation corrected to individualized BSA (ml/min)

Classification systems		CKD-EPI 2021					
		1	2	3	4	5	Total
CKD-EPI 2021 corrected to i-BSA	1	2156	682	0	0	0	2838
	2	264	1656	49	0	0	1969
	3	0	53	87	0	0	140
	4	0	0	0	4	0	4
	5	0	0	0	0	0	1
	Total	2420	2391	136	4	1	4952

CKD, chronic kidney disease; CKD-EPI, chronic kidney disease-epidemiology collaboration; i-BSA, individualized body surface area in m^2^.

### Outcome analyses according to CKD definitions

**Figure 2:**
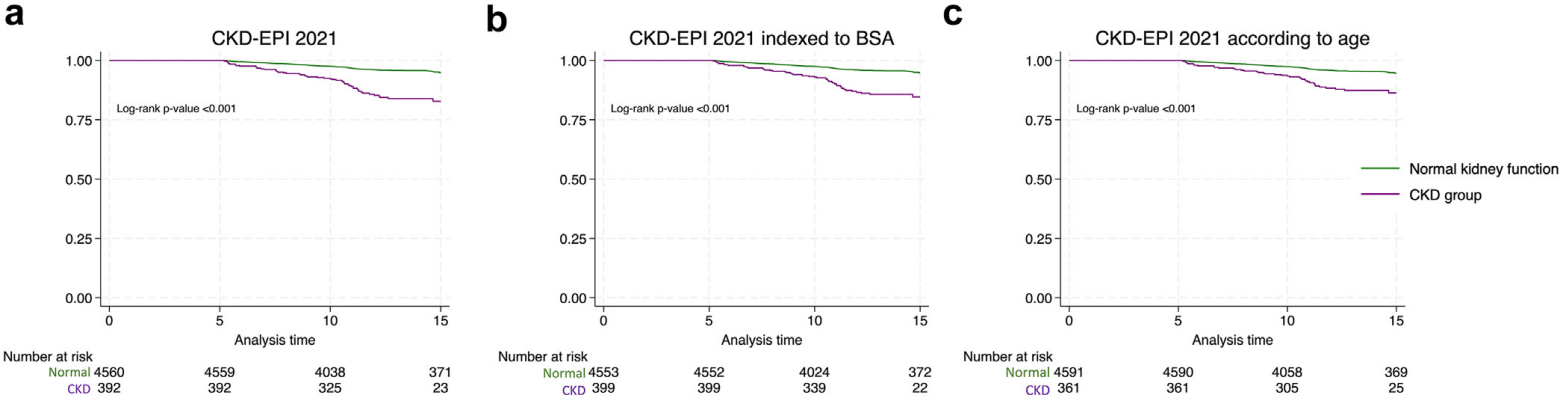
Kaplan Meier survival curve for combined outcome (RRFD and global mortality) according to the 3 CKD definitions (n = 4952). Survival curves comparing patients with normal kidney function (green) with those with CKD (purple) over time for the 3 different CKD definitions: (a) classical CKD-EPI 2021 equation in the first graph, (b) CKD-EPI 2021 corrected to individualized body surface area (i-BSA) in the second graph, (c) and age-stratified CKD-EPI 2021 in the last graph. Differences are significant (P < 0.001). CKD, chronic kidney disease; CKD-EPI, CKD Epidemiology Collaboration; RRFD, rapid renal function decline.

In Figure 2a to c we present, the Kaplan-Meier survival analyses for the 3 different classifications, separately. Consistently, we observed in the CKD-defined group compared with the non-CKD group, an increased rate of the composite outcome, combining RRFD and overall mortality, with all log-rank *P*-values < 0.001. Univariate analysis yielded in each of the 3 CKD-group HRs of 3.7 (95% CI: 2.7–5.1) for the classical CKD-EPI 2021 classification, 3.2 (95% CI: 2.3–4.4) when corrected to i-BSA, and 2.7 (95% CI: 1.9–3.9) for age-stratified CKD-EPI 2021 model. Proportional hazards assumptions for Cox model were confirmed using Schoenfeld residuals.

In Table 4, we show the adjusted HRs in the multivariate Cox regression analysis: HR 2.2 (95% CI: 1.6–3.1) for CKD-EPI 2021 classification, 2.1 (95% CI: 1.5–2.9) when corrected for i-BSA (ml/min), and 1.6 (95% CI: 1.1–2.4) stratified for age CKD-EPI 2021 model. The full analysis showing the HR for each variable is available as Supplementary Table S1A to C.

**Table 4:** Univariate and multivariable Cox regression for combined outcome (RRFD and global mortality) according to the 3 CKD definitions

CKD Definition	Univariate	Multivariable^a^
	HR [95% CI]	HR [95% CI]
CKD-EPI 2021	3.73 [2.73–5.09]^b^	2.23 [1.59–3.12]^b^
CKD-EPI 2021 corrected to i-BSA	3.21 [2.33–4.42]^b^	2.06 [1.46–2.90]^b^
CKD-EPI 2021 stratified to age	2.74 [1.93–3.88]^b^	1.64 [1.13–2.38]^b^

BMI, body mass index; CI, confidence interval; CKD, chronic kidney disease; HR, hazard ratio; i-BSA, individualized body surface area in m^2^; RRFD, rapid renal function decline.

a Multivariable regression adjusting for sex, hypertension, diabetes, BMI, treated dyslipidemia, smoking, and history of cardiovascular events.

b All hazard ratios had statistical significance with a *P* value < 0.001.

**Figure 3:**
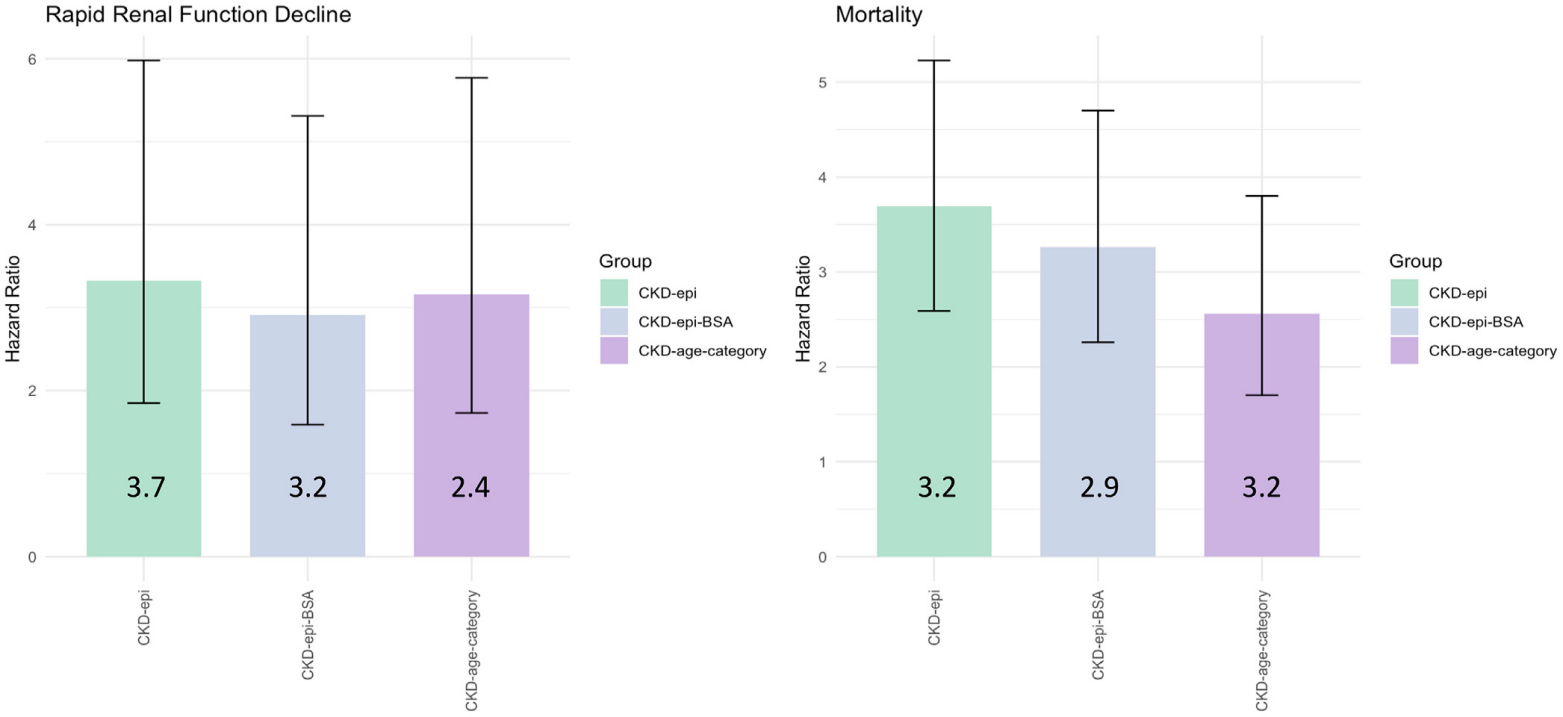
Compounds of composite outcome for the 3 CKD definitions (*n* = 4952). The bar graphs represent hazard ratios of univariate analysis with 95% confidence interval (error bars) for rapid renal function decline (RRFD) and mortality across the 3 CKD definition groups: CKD-EPI 2021 (green), CKD-EPI 2021 corrected to individualized BSA (blue), and CKD-EPI 2021 stratified to age (purple). No significant differences were observed between the groups for either outcome. BSA, body surface area; CKD, chronic kidney disease; CKD-EPI, CKD Epidemiology Collaboration.

In Figure 3, we illustrate the separated analyses for RRFD and global mortality corroborating these results.

**Figure 4:**
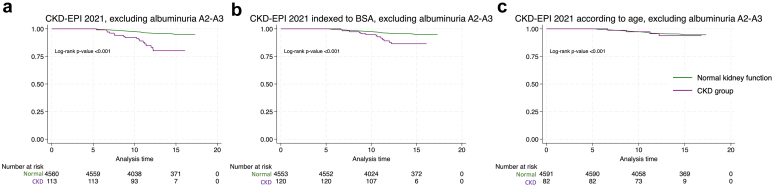
Kaplan Meier survival curve for combined outcome depending on CKD definition after exclusion of albuminuric patients (*n* = 4673). Survival curves comparing patients with normal kidney function (blue) to those with CKD (red) over time for the 3 different CKD definitions after exclusion of patients with significative albuminuria (>30 mg/g): classical CKD-EPI 2021 equation in the first graph, CKD-EPI 2021 corrected to individualized to BSA in the second graph, and age-adjusted CKD-EPI 2021 in the last graph. Differences are significant (*P* < 0.001). CKD, chronic kidney disease; CKD-EPI, CKD Epidemiology Collaboration.

By excluding patients with albuminuria, and thus considering only eGFR for the outcome occurrence, results were similar (Figure 4).

### CKD Thresholds in Younger and Older Patient Subgroup

In the younger subset of our cohort, aged < 40 years, 24 patients (0.5%) were diagnosed with CKD according to the new age-specific criteria, with no adverse outcomes observed. Conversely, for patients aged > 65 years, 55 individuals (1.1%) were reclassified from CKD to non-CKD with the revised definition, from which 13 (23.6%) presented an adverse outcome (12 deaths and 1 RRFD).

**Figure 5:**
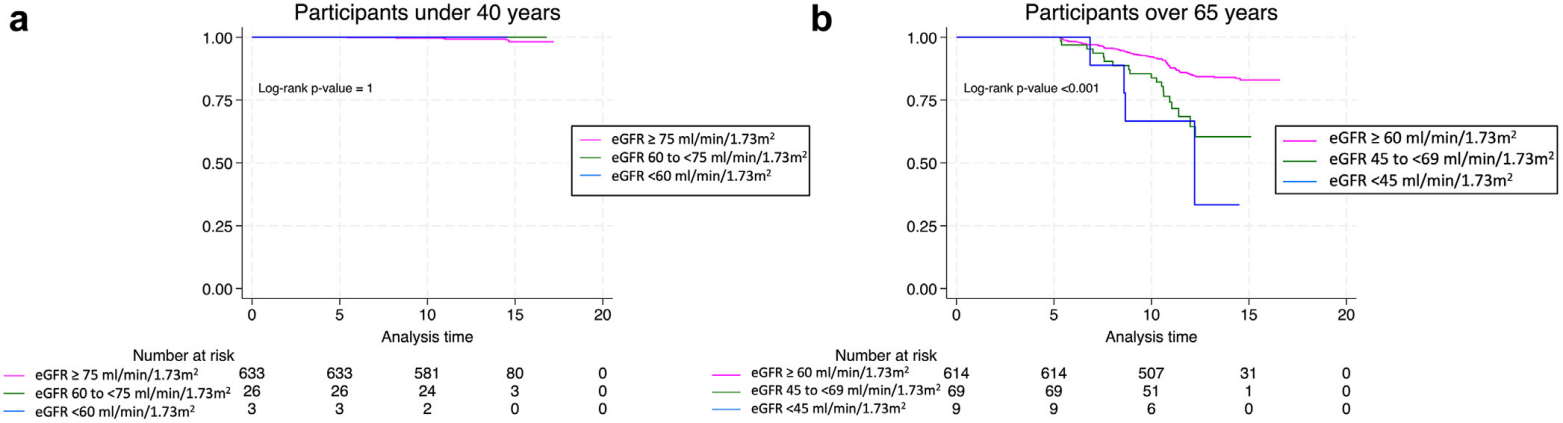
Kaplan Meier survival curve for combined outcome (RRFD and global mortality) in age groups, stratified in GFR categories. Survival curves comparing patients with different eGFR thresholds over time for patients (a) aged < 40 years, and (b) aged ≥65 years. The first graph shows that, for patient aged < 40 years, the survival curves for CKD-threshold at 70 or at 60 ml/min per 1.73 m^2^ are overlapping. The second graph illustrates in brown, the survival curve of the actual cutoff used to define CKD, namely 60 ml/min per 1.73 m^2^; and in yellow, a diminished survival if CKD-threshold is lowered at 45 ml/min per 1.73 m^2^. Log-rank test confirms that curves are significantly different in both graph (*P* < 0.001). CKD, chronic kidney disease; eGFR, estimated GFR; GFR, glomerular filtration rate; RRFD, rapid renal function decline.

The survival curves for patients aged < 40 years indicate similar survival probabilities for all eGFR thresholds of ≥ 75 ml/min per 1.73 m^2^ and 60 to 74 ml/min per 1.73 m^2^ and < 60 ml/min per 1.73 m^2^ (Figure 5a). This suggests that in this younger cohort, the difference in eGFR within this range does not significantly affect survival outcomes within the duration of this study.

Conversely, the survival curves for patients aged ≥ 65 years demonstrate a more distinct separation between eGFR thresholds, with the ≥ 60 ml/min per 1.73 m^2^ group exhibiting better survival outcomes (Figure 5b). The curves for the 45 to 59 ml/min per 1.73 m^2^ and < 45 ml/min per 1.73 m^2^ thresholds, however, show an overlap, indicating no substantial difference in survival between these groups. The limited number of events in the lowest eGFR stratum may restrict the statistical reliability of these findings.

## Discussion

According to this Caucasian population-based study including 4952 participants, the outcomes of CKD did not substantially differ depending on the 3 CKD definitions based on the standard, corrected to i-BSA, and age-stratified CKD-EPI 2021 equations.

Indeed, the combined risk of increased mortality and rapid decline in renal function was increased in patients with CKD, with no significant difference between the 3 groups. After adjustment to confounders, the HRs were 2.2 (95% CI: 1.6–3.1) for the standard definition; 2.1 (95% CI: 1.5–2.9), if corrected on i-BSA; and 1.6 (95% CI: 1.1–2.4) if stratified by age. Moreover, upon separate examination of RRFD and mortality, no disparities were evident.

Reclassification of participants based on i-BSA generally shifted individuals (15%) to lower-risk CKD categories. This could be interpreted as a potential underestimation of risk by the new definitions or an overestimation by the currently used CKD-EPI 2021 equation. The analysis revealed that 264 people (5% of the cohort) were reassigned to higher-risk CKD categories, whereas 731 participants (15%) were moved to lower-risk categories (stages 1 and 2). Interestingly, no patients were reclassified as not having CKD, or vice versa. Thus, the probability of overlooking or overestimating patients remains constant. Nevertheless, these nuanced adjustments provoke reflection on the i-BSA correction.

Age subgroup analysis revealed that 24 participants (0.5%) aged < 40 years were reclassified as having CKD under the new age-adapted CKD definition but did not develop adverse outcomes, such as RRFD or death. Furthermore, survival analysis indicated that younger patients maintained comparable survival rates across both conventional (< 60 ml/min per 1.73 m^2^) and higher (< 75 ml/min per 1.73 m^2^) eGFR thresholds, suggesting minimal impact of changing threshold in this age range. Conversely, among those aged > 65 years, 55 participants (1.1%) were reclassified as not having CKD, yet 13 of them (24%) experienced adverse outcomes, namely 12 deaths and 1 RRFD. Thus, these patients would have been missed during CKD identification using the age-stratified definition. These findings suggest the unnecessary need for an age-stratification for eGFR thresholding in such a general population.

The lack of observed benefit in using age-based or i-BSA CKD definition in terms of survival for our combined outcome calls into question the current trend toward personalized GFR thresholds based on age and i-BSA. This finding is particularly intriguing given the increasing body of literature suggesting that physiological decline in GFR with age does not necessarily indicate pathological changes. In fact, a growing consensus in the clinical community emphasizes the importance to differentiate between changes in kidney structure and function resulting from early morbidity and mortality, and the anatomical and functional kidney adaptations observed in the context of normal, healthy aging.^3^^,^^16^^,^^17^

Indeed, the study by Liu *et al.*^5^ demonstrated that lowering the eGFR threshold for diagnosing CKD in the elderly can reduce the number of individuals diagnosed with this condition. Those reclassified as having normal kidney function did not exhibit an increased risk of death or kidney failure. Specifically, the authors observed that 75% of the cohort, consisting of individuals aged ≥ 65 years with an eGFR between 45 and 59 ml/min per 1.73 m^2^ and either normal or mild albuminuria, faced risks of renal failure and death comparable to those without CKD.^5^^,^^18^ These findings align with results from another study conducted in Iceland.^17^ It showed that age-based reclassification led to 50% decrease in the overall prevalence of CKD individuals over 65 and confirmed that the standardized mortality ratio remained stable in those aged over 65 with an eGFR of 45 to 60 ml/min per 1.73 m^2^, who had no proteinuria or diagnosis of primary kidney disease. These results contrast with our findings, because reclassification has shown a significant number of patients who are newly considered to have normal kidney function under the age-adapted definition, but have experienced severe renal outcome.

We could attribute the lack of significant age stratification effects in our study to the average age of our cohort being 52 years and the limited number of patients aged < 40 and > 65 years, where age-based reclassification is likely to have a greater impact. Indeed, in our study, 51% of patients were reclassified as having a normal kidney function, compared with 75% in Liu *et al.*^5^'s study. We could also think that in the younger group, a longer follow-up would be necessary to observe any adverse event. Indeed, the number of events in those aged < 40 years was quite low.

Furthermore, our findings are corroborated by a meta-analysis conducted by Hallan *et al.*,^19^ which included over 2 million participants from 33 general population or high-cardiovascular-risk cohorts and 13 CKD cohorts across Asia, Australasia, Europe, and North and South America. It showed that among patients aged > 65 years, HR for mortality was higher with an eGFR of 45 ml/min per 1.73 m^2^ compared with 80 ml/min per 1.73 m^2^, with an HR of 1.59 (95% CI: 1.42–1.77).^19^

### Strengths and Limitations

Our study has several strengths, including its large population-based design in a geographically defined population with universal access to health care. In addition, our study is original in comparing 3 definitions that use different eGFR criteria.

Our study also has some limitations. First, the study source population was only Caucasian aged 35 to 75 years, and this may limit the generalizability of our findings to populations with different ethnic composition and older population. Second, the duration of our study may have been insufficient to detect kidney disease progression and mortality outcomes in younger individuals captured by the age-adapted definition only. Longer follow-up studies are needed to compare outcomes. Prospective studies are needed to clarify the extent to which any possible association may be the result of aging or the eGFR decline that accompanies aging. In addition, we did not have cystatin C measured to improve the eGFR calculation; however, we would expect the changes to be similar in the 3 equations groups, and therefore this lack of precision should not have an impact in our results. Finally, the CKD definition was based on a single eGFR measurement at each time point, which cannot completely exclude the possibility of an acute kidney injury. However, the likelihood of acute kidney injury is minimized because the participants were not hospitalized and showed no acute symptoms at the time of evaluation.

Prospective studies with extended follow-up periods are needed to further understand the long-term effects of age and i-BSA-based reclassification of CKD. Moreover, exploring other biomarkers and risk factors could deepen our understanding of CKD progression and lead to more effective management strategies.

### Implications for Clinical Practice

Our study has implications for clinical practice, health policy, and research. “Less is more” may be the best option. In fact, the simplification of GFR interpretation has the advantage of limiting the complexity of diagnosis and allowing every general practitioner to easily understand and apply it.

In conclusion, although age stratification and i-BSA correction of GFR are conceptually appealing, their limited impact on key outcomes in our study suggests that the current CKD classification system may still be appropriate for clinical use in most of the Caucasian population. To note, the actual Kidney Disease Improving Global Outcomes 2024 guidelines have kept for the moment the standard equation without proposing an adapted one according to age or i-BSA. Our findings contribute to a growing body of evidence indicating that a more nuanced approach, incorporating various clinical factors, may be necessary to improve outcomes for patients with CKD.

## Disclosure

All the authors declared no competing interests.

The results presented in this paper have not been published previously in whole or part, except in abstract format.

## Supplementary Material

Supplementary File (PDF) Table S1. Multivariable Cox regression for combined outcome (RRFD and global mortality) according to the 3 CKD definitions (*N* = 5052): (A) classical CKD-EPI 2021 definition, (B) CKD-EPI 2021 corrected to individualized BSA, and (C) CKD-EPI 2021 stratified by age groups. **STROBE Checklist.**

## Footnotes

Supplementary File (PDF)
**Table S1.** Multivariable Cox regression for combined outcome (RRFD and global mortality) according to the 3 CKD definitions (*N* = 5052): (A) classical CKD-EPI 2021 definition, (B) CKD-EPI 2021 corrected to individualized BSA, and (C) CKD-EPI 2021 stratified by age groups.
**STROBE Checklist.**
