# Supplementary material for: Impact of Different CKD Definitions on Long-Term Renal Function and Mortality in a Population-Based Cohort Study
Source: Kidney Int Rep. 2024 Nov 28;10(2):386–95. doi: 10.1016/j.ekir.2024.11.027 (PMC11843124; doi:10.1016/j.ekir.2024.11.027)
Supplement: Supplementary File (PDF) — Table S1. Multivariable Cox regression for combined outcome (RRFD and global mortality) according to the 3 CKD definitions (N = 5052): (A) classical CKD-EPI 2021 definition, (B) CKD-EPI 2021 corrected to individualized BSA, and (C) CKD-EPI 2021 stratified by age groups. STROBE Checklist. [file mmc1.pdf]

Supplementary figures and tables

Supplementary table 1 (a-b-c): Multivariable cox regression for combined outcome (RRFD and global mortality) according to the three CKD definitions (n=5052): classical CKD-EPI 2021 definition (a), CKD-EPI 2021 indexed to BSA (b), and CKD-EPI 2021 stratified by age groups (c).

a.	HR	95% CI	P value
**eGFR – classic**	2.23	1.59 – 3.12	<0.001
Gender	1.21	0.92 – 1.59	0.161
Hypertension	2.35	1.74 – 3.19	<0.001
Diabetes	1.95	1.33 – 2.86	0.001
BMI	1.04	1.01 – 1.07	0.007
Dyslipidemia	0.87	0.60 – 1.26	0.454
Smoking	1.52	1.15 – 2.03	0.004
Prior CV events			
-MI	1.62	0.99 – 2.66	0.054
-Angina pectoris	1.21	0.70 – 2.11	0.496
-Heart failure	1.23	0.69 – 2.17	0.480
-Stroke	2.47	1.69 – 3.61	<0.001

b.	HR	95% CI	P value
**eGFR – BSA indexed**	2.01	1.46 – 2.90	<0.001
Gender	1.21	0.93 – 1.59	0.161
Hypertension	2.38	1.34 – 2.89	0.001
Diabetes	1.97	1.60 – 2.92	<0.001
BMI	1.04	1.01 – 1.08	0.004
Dyslipidemia	0.87	0.60 – 1.27	0.479
Smoking	1.51	1.14 – 2.01	0.004
Prior CV events			
-MI	1.63	0.99 – 2.66	0.053
-Angina pectoris	1.21	0.70 – 2.11	0.498
-Heart failure	1.24	0.70 – 2.19	0.460
-Stroke	2.50	1.71 – 3.65	<0.001

c.	HR	95% CI	P value
**eGFR – age stratified**	1.64	1.13 – 2.38	0.009
Gender	1.19	0.91 – 1.56	0.206
Hypertension	2.44	1.81 – 3.30	<0.001
Diabetes	2.07	1.41 – 3.04	<0.001
BMI	1.04	1.01 – 1.07	0.005
Dyslipidemia	0.88	0.60 – 1.28	0.498
Smoking	1.50	1.13 – 2.00	0.005
Prior CV events			
-MI	1.64	1.01 – 2.68	0.048
-Angina pectoris	1.19	0.68 – 2.07	0.544
-Heart failure	1.31	0.74 – 2.31	0.358
-Stroke	2.58	1.77 – 3.77	<0.001

Abbreviations: Hazard Ratio (HR); Confidence Interval (CI); estimated glomerular filtration rate (eGFR); Body mass index (BMI); Cardiovascular (CV); Myocardial infraction (MI); Body surface aera (BSA) in m^2

STROBE Statement—checklist of items that should be included in reports of observational studies

	Item No.	Recommendation	Page No.	Citations
**Title and abstract**	1	(*a*) Indicate the study's design with a commonly used term in the title or the abstract	1	*"Impact of different chronic kidney disease definitions on long term renal function and mortality in a population-based cohort study."*
		(*b*) Provide in the abstract an informative and balanced summary of what was done and what was found	2	See abstract.
**Introduction**				
Background/rationale	2	Explain the scientific background and rationale for the investigation being reported	3	See introduction.
Objectives	3	State specific objectives, including any prespecified hypotheses	4	*"In the present study, we aimed to evaluate CKD reclassification within a Swiss general population cohort by applying 3 different equations: the conventional fixed thresholds used in CKD-EPI 2021 equation, individualized-BSA eGFR, and age-specific eGFR thresholds. We then studied their effects on the prediction of rapid renal function decline (RRFD) and mortality. We hypothesize that the use of age-specific or individualized BSA criteria will result in a decreased CKD prevalence and a reduced mortality and RRFD over an up to 15-years follow-up period."*
**Methods**				
Study design	4	Present key elements of study design early in the paper	5	*"The dataset for this investigation was derived from the CoLaus/PsyCoLaus study, a general population-based cohort study in Lausanne, Switzerland with follow-up assessments conducted at 5, 10 and 15 years."*
Setting	5	Describe the setting, locations, and relevant dates, including periods of recruitment, exposure, follow-up, and data collection	5	*"The baseline survey took part between 2003 and 2006, encompassing a cohort of 6733 individuals aged 35 to 75, of whom 6184 were Caucasian. Detailed methodologies concerning participant selection, evaluation protocols, and clinical data acquisition have been documented in a previous study[7]. Briefly, recruitment occurred within the city of Lausanne, which had a population of*

				117,161 residents at that time. The municipal registry provided a complete list of 56,694 residents within the age range (35 to 75), from which a simple, non-stratified random sampling method was used to contact 35% of this population through mailed correspondences."
Participants	6	(*a*) *Cohort study*—Give the eligibility criteria, and the sources and methods of selection of participants. Describe methods of follow-up	5	*"Inclusion criteria stipulated Caucasian ethnicity and the ability to provide written informed consent. The response rate for this randomly selected sample was 41%. Patients with missing albuminuria and normal eGFR according to one of the three CKD definitions, or patients with no follow-up either at 5, 10 or 15 years were excluded. Ethical approval for the study was granted by the local ethics committee, and participants provided all written informed consent form."*
Variables	7	Clearly define all outcomes, exposures, predictors, potential confounders, and effect modifiers. Give diagnostic criteria, if applicable	7	*"The primary outcome was a combined incidence of rapid renal function decline (RRFD) and global mortality, defined as all-cause mortality, over the follow-up duration. Following KDIGO guidelines, which defined rapid renal function decline as a sustained eGFR reduction of more than 5 ml/min/1.73m2 per year [15], we defined RRFD as a decrease of more than 25 mL/min/1.73 m² at 5 years, more than 50 mL/min/1.73 m² at 10 years, and more than 75 mL/min/1.73 m² at 15 years, comparing baseline value to follow-up measurement. These parameters were aggregated into a composite outcome to both represent the adverse progression that effective nephroprotection could potentially prevent and to increase the total number of events observed. The secondary outcomes were RRFD and mortality analyzed separately."*
Data sources/ measurement	8*	For each variable of interest, give sources of data and details of methods of assessment (measurement). Describe comparability of assessment methods if there is more than one group	5-6	See "Survey and clinical data acquisition" and "Laboratory data" sections.
Bias	9	Describe any efforts to address potential sources of bias	5-6	*For example:*<br>*"a general population-based cohort study in Lausanne" (page 5)*<br>*"standardized questionnaires" (page 5)*

				"Blood pressure measurements were taken three times on the left arm of seated participant using a clinically validated automatic oscillometric device." (page 6)<br>*"Serum and urine creatinine levels were measured using the IDMS-traceable Jaffe kinetic compensated method by Roche Diagnostics, Switzerland, which guarantees accuracy and reliability, with maximum intra- and inter-batch coefficients of variation ranging from 2.9% to 0.7%." (page 6)*
Study size	10	Explain how the study size was arrived at	5	*"The baseline survey took part between 2003 and 2006, encompassing a cohort of 6733 individuals aged 35 to 75, of whom 6184 were Caucasian. Detailed methodologies concerning participant selection, evaluation protocols, and clinical data acquisition have been documented in a previous study[7]."*

Quantitative variables	11	Explain how quantitative variables were handled in the analyses. If applicable, describe which groupings were chosen and why	7	*"We categorized patients using sequentially three CKD-definition, each including albuminuria yet distinguished by varying eGFR thresholds: (1) eGFR < 60 ml/min/1.73m2; (2) eGFR< 60 ml/min corrected to individualized BSA (i-BSA); (3) age-stratified eGFR with CKD defined as eGFR was <75 ml/min/1.73m2 for individuals < 40 years old, < 60 ml/min/1.73m2 for ages 40-65, and < 45 ml/min/1.73m2 for those > 65, according to the previous studies definitions [14]. "*
Statistical methods	12	(*a*) Describe all statistical methods, including those used to control for confounding	7-8	*"Descriptive analysis presented continuous variables as mean ± standard deviation (SD) or median with interquartile range, according to their distribution. Categorical variables are expressed as counts and percentages. Separate models using the three CKD definition – namely the classical CKD-EPI 2021, i-BSA CKD, and age-stratified CKD definition – were deployed to examine their influence on the primary composite outcome, which includes mortality and RRFD. Kaplan-Meier estimates were used for assessing survival probabilities, while Cox proportional hazards models analyzed time-to-event data. Survival curves were compared using the log-rank test. Cox models provided Hazard Ratios (HRs), 95% Confidence Intervals (CI), and associated P values. The assumption of hazard proportionality was visually verified using Schoenfeld residuals (log-minus-log survival plots against time).*

				Selection of covariates in multivariable Cox analyses was based on existing scientific literature to identify potential confounders. The Cox proportional hazards model included variables such as diabetes, sex, body mass index (BMI), and arterial hypertension, treated dyslipidemia, smoking, and prior cardiovascular events."
		(*b*) Describe any methods used to examine subgroups and interactions	8	*"In secondary analyses, RRFD and global mortality were examined separately. Additionally, Kaplan-Meier survival analysis was used to evaluate the prognostic implication of different eGFR CKD thresholds in two age-defined patient subgroups. For individuals younger than 40 years, eGFR-strata were ≥75, 60-74 and <60 ml/min/1.73 m2; for those aged 65 years and older, eGFR-strata were ≥60, 45-60 and <45 ml/min/1.73 m2, allowing for a comparative assessment of survival outcomes across these eGFR thresholds."*
		(*c*) Explain how missing data were addressed	8	*"Missing data were assumed to occur at random, and variable was excluded if missing data exceeded 5%."*
**Results**				
Participants	13*	(a) Report numbers of individuals at each stage of study—eg numbers potentially eligible, examined for eligibility, confirmed eligible, included in the study, completing follow-up, and analysed	8	*"Among the 6184 Caucasian individuals of the CoLaus study, 4952 (80.1%) were included in our analyses. Figure 1 illustrates the study's flowchart"*
		(b) Give reasons for non-participation at each stage	8	*"The exclusion criterion impacting the study the most was the absence of follow-up."*
		(c) Consider use of a flow diagram	8	See Figure 1.
Descriptive data	14*	(a) Give characteristics of study participants (eg demographic, clinical, social) and information on exposures and potential confounders	9	See Tables 1 and 2.
		(b) Indicate number of participants with missing data for each variable of interest		See Tables 1 and 2.
		(c) *Cohort study*—Summarise follow-up time (eg, average and total amount)	9	*"The median follow-up duration was 12.7 years (SD ±2.9; range 4.9-17.3)."*

Outcome data	15*	*Cohort study*—Report numbers of outcome events or summary measures over time	10	*"Within the cohort, 72 participants (1.4%) experienced RRFD and 170 (3.4%) died, from whom 11 (0.2%) experienced both outcomes."*
Main results	16	(*a*) Give unadjusted estimates and, if applicable, confounder-adjusted estimates and their precision (eg, 95% confidence interval). Make clear which confounders were adjusted for and why they were included	10	"Table 4 shows the adjusted hazard ratios in the multivariate Cox regression analysis: HR 2.2 (95% CI: 1.6-3.1) for CKD-EPI 2021 classification, 2.1 (95% CI: 1.5-2.9) when corrected for i-BSA (ml/min) and 1.6 (95% CI: 1.1-2.4) stratified for age CKD-EPI 2021 model. The full analysis showing the HR for each variable is available as supplementary Table 1 (a-b-c)." (Page 10)<br>And: *"Selection of covariates in multivariable Cox analyses was based on existing scientific literature to identify potential confounders. The Cox proportional hazards model included variables such as diabetes, sex, body mass index (BMI), and arterial hypertension, treated dyslipidemia, smoking, and prior cardiovascular events." (page 8)*
		(*b*) Report category boundaries when continuous variables were categorized	7-9	See Methods
		(*c*) If relevant, consider translating estimates of relative risk into absolute risk for a meaningful time period	9	*"Within the cohort, 72 participants (1.4%) experienced RRFD and 170 (3.4%) died, from whom 11 (0.2%) experienced both outcomes."*

Other analyses	17	Report other analyses done—eg analyses of subgroups and interactions, and sensitivity analyses	10-11	See "CKD thresholds in younger and older patient subgroup" section.
**Discussion**				
Key results	18	Summarise key results with reference to study objectives	11-12	*"According to this Caucasian population-based study including 4952 participants, the outcomes of CKD did not substantially differ depending on the three CKD definitions based on standard, corrected to i-BSA, and age-stratified CKD-EPI 2021 equations (…)."*
Limitations	19	Discuss limitations of the study, taking into account sources of potential bias or imprecision. Discuss both direction and magnitude of any potential bias	13	See "*Strengths and limitations*" section.
Interpretation	20	Give a cautious overall interpretation of results considering objectives, limitations, multiplicity of analyses, results from similar studies, and other relevant evidence	12-13	See "*Discussion*" section.

Generalisability	21	Discuss the generalisability (external validity) of the study results	13-14	*"First, the study source population was only Caucasian aged 45 to 75, and this may limit the generalizability of our findings to populations with different ethnic composition and older population."*
**Other information**				
Funding	22	Give the source of funding and the role of the funders for the present study and, if applicable, for the original study on which the present article is based	16	*"The CoLaus/PsyCoLaus study was supported by research grants from GlaxoSmithKline, the Faculty of Biology and Medicine of Lausanne, the Swiss National Science Foundation (grants 33CSCO-122661, 33CS30-139468, 33CS30-148401, 33CS30_177535, 3247730_204523 and 320030_220190) and the Swiss Personalized Health Network (grant 2018DRI01).*<br><br>*L.B. is supported by a grant from the Swiss National Science Foundation (PZ00P3_208670/1) and a grant Projet de Recherche et développement (PRD) from the University hospital of Geneva (PRD 19-2018). S.d.S. is supported by a grant from the Swiss National Science Foundation (SNSF 320030_204187). D.D. is the recipient of a grant from the Swiss National Science Foundation (32350_214547). A.H. is the recipient of a grant from the Swiss National Science Foundation (323530_221874). "*

*Give information separately for cases and controls in case-control studies and, if applicable, for exposed and unexposed groups in cohort and cross-sectional studies.

Note: An Explanation and Elaboration article discusses each checklist item and gives methodological background and published examples of transparent reporting. The STROBE checklist is best used in conjunction with this article (freely available on the Web sites of PLoS Medicine at http://www.plosmedicine.org/, Annals of Internal Medicine at http://www.annals.org/, and Epidemiology at http://www.epidem.com/). Information on the STROBE Initiative is available at www.strobe-statement.org.
